# Risk of SARS-CoV-2 infection following initial COVID-19 vaccination: Population-based cohort study

**DOI:** 10.1371/journal.pone.0273903

**Published:** 2022-10-20

**Authors:** Mina Tadrous, Hannah Chung, Siyu Men, Cherry Chu, Tonya Campbell, David N. Juurlink, Jeffrey C. Kwong, J. Michael Paterson, Tara Gomes

**Affiliations:** 1 Leslie Dan Faculty of Pharmacy, University of Toronto, Toronto, ON, Canada; 2 ICES, Toronto, ON, Canada; 3 Women’s College Research Institute, Women’s College Hospital, Toronto, ON, Canada; 4 Li Ka Shing Knowledge Institute of St. Michael’s Hospital, Unity Health Toronto, Toronto, ON, Canada; 5 Institute of Health Policy, Management and Evaluation, University of Toronto, Toronto, ON, Canada; 6 Sunnybrook Research Institute, Toronto, ON, Canada; 7 Public Health Ontario, Toronto ON, Canada; 8 Dalla Lana School of Public Health, University of Toronto, Toronto, ON, Canada; 9 Centre for Vaccine Preventable Diseases, University of Toronto, Toronto, ON, Canada; 10 Department of Family and Community Medicine, University of Toronto, Toronto, ON, Canada; 11 University Health Network, Toronto, ON, Canada; DM Wayanad Institute of Medical Sciences, INDIA

## Abstract

**Background:**

Anecdotally there are reports of newly diagnosed SARS-CoV-2 infection shortly after vaccination. This has led some to speculate that vaccination itself might inadvertently increase the short-term risk of COVID potentially due to airborne spread at mass vaccination clinics or relaxation of precautions following vaccination. We explored whether receipt of vaccination was associated with a short-term increase in the risk of being diagnosed with COVID-19 and if differences exist between vaccination settings.

**Methods:**

We conducted a cohort study in Ontario, Canada to compare the risk of SARS-CoV-2 infection within 21 days of receiving a first vaccination, according to the setting in which vaccines were administered between March 1, 2021 and May 6, 2021. We used linked population-wide vaccination, laboratory testing, and health administrative databases. We created a 1:1 matched comparison group of unexposed individuals. We reported the overall risk of infection calculated at 3, 7, 10, 14, 18, and 21 days. This was completed overall and by setting of vaccine receipt.

**Results:**

We identified 4,798,430 Ontario residents who received their first dose of a COVID-19 vaccine. In the primary analysis, the rate of SARS-CoV-2 infection was significantly lower among vaccine recipients vs non-recipients at all the post-vaccination time points. Analysis stratified by vaccination setting found that mass vaccination clinics, pharmacies, and physician offices were consistent with the main findings. Individuals who received their first vaccine dose in congregate residential settings had a higher rate of SARS-CoV-2 infection at 7 days (HR 1.35, 95% CI 1.00–1.83) and 10 days (HR 1.49, 95% CI 1.03–2.15).

**Conclusion:**

In this population-based cohort study, we found that there was no increased risk of SARS-CoV2 infection after vaccination suggesting no broad transmission of disease at time of vaccination. Some evidence of increased risk among those vaccinated in congregate settings, highlighting the need to consider opportunities for supporting safe vaccine administration in these settings. Given ongoing and future immunization programs, the results support the need for continued vigilance during any mass vaccination processes and education regarding the delayed nature of protection following vaccination.

## Introduction

The SARS-CoV-2 pandemic has had major clinical, social and economic impacts globally. Vaccination against SARS-CoV-2 is a key element of the current strategy to end the pandemic. Despite the ongoing campaign reaching over 85% of Canadians, successive waves of infection have continued around the globe. Currently initiatives to administer third dose boosters are ongoing with potential plans for fourth doses in high-risk patients and potential periodic annual boosters are being discussed [1,2].

Existing COVID-19 vaccines have been shown to be efficacious at reducing SARS-CoV-2 infection beginning approximately 14 days after the first dose, as well as dramatically reducing the risk of hospitalization and death [3–6]. However, anecdotally some patients newly diagnosed with COVID-19 report the onset of symptoms shortly after vaccination. This has led some to speculate that vaccination itself might inadvertently increase the short-term risk of COVID. Such an association is plausible on account of airborne spread at mass vaccination clinics, relaxation of precautions such as hand washing, social distancing, and masking following vaccination, or both. In light of this, and because an ongoing need for vaccination programs owing to the likely emergence of new variants, we explored whether receipt of vaccination was associated with a short-term increase in the risk of being diagnosed with COVID-19.

## Methods

### Study design and population

We conducted a population-based cohort study in Ontario, Canada to compare the risk of SARS-CoV-2 infection within 21 days of receiving a first vaccination, according to the setting in which vaccines were administered. We included all Ontarians aged 18 years or older who were eligible for the universal Ontario Health Insurance Plan (OHIP) and vaccinated with a valid OHIP number between March 1, 2021 and May 6, 2021. We excluded individuals who tested positive for SARS-CoV-2 prior to March 1, 2021, as well as those who resided in a long-term care facility or died prior to the start of the observation period.

### Data sources and definitions

We obtained data from ICES (formerly Institute for Clinical Evaluative Sciences, www.ices.on.ca), which houses comprehensive, population-based databases for SARS-CoV-2 laboratory testing, SARS-CoV-2 public health surveillance, COVID-19 vaccination, and healthcare system use for all Ontario residents eligible for OHIP (approximately 14.6 million people). The details of the data sources have been described previously [7]. We obtained information regarding COVID-19 vaccination status, including date of administration, setting of administration (congregate residential setting, hospital, mass clinic, physician office, pharmacy or other setting), and vaccine dose number, from COVaxON, a centralized provincial COVID-19 vaccine information system.

We collected data on laboratory-confirmed SARS-CoV-2 infection detected by real-time reverse transcription polymerase chain reaction (RT-PCR) from the Ontario Laboratories Information System (OLIS). We used the OHIP Registered Persons Database (RPDB) to ascertain age, sex, and postal code of residence of study subjects, and used the Canada Post Postal Code Conversion File and 2016 Statistics Canada Census to identify public health region of residence, urban vs. rural residence status, and neighbourhood income quintile. We ascertained comorbidities from a variety of linked administrative databases including the OHIP Claims History Database, which captures physician service claims; the Canadian Institute for Health Information (CIHI) Discharge Abstract Database, which captures procedures and diagnoses for all acute hospital admissions; and the CIHI National Ambulatory Care Reporting System, which contains records for all emergency department admissions [7]. These datasets were linked using unique encoded identifiers and analyzed at ICES. The use of the data in this project was authorized under Section 45 of Ontario’s Personal Health Information Protection Act and did not require review by a Research Ethics Board.

### Exposure, outcome, and covariates

We defined vaccine exposure as receipt of a first dose of a Health Canada authorized COVID-19 vaccine between March 1, 2021 and May 6, 2021, with the date of vaccine administration serving as the study index date. March 1, 2021 marks the period of increasing access to COVID-19 vaccines in Ontario following the launch of provincial mass vaccination campaigns; prior to this date vaccination was limited to healthcare workers and those living in nursing homes. We created a 1:1 matched comparison group of unexposed individuals by identifying individuals who did not receive a COVID-19 vaccine as of each vaccinated individual’s index date or during the 21-day follow-up period using a two-step matching process. First, we matched each exposed individual to all unexposed individuals of the same sex who resided in the same Statistics Canada Census Tract (small, relatively stable geographic areas that comprise 2,000–8,000 residents) as of March 1, 2021. Second, after assigning the exposed individual’s index date to all of the initially-matched unexposed individuals, we narrowed the pool of unexposed individuals by matching further on age at index date (+/- 5 years) and the number of SARS-CoV-2 tests within 90 days prior to the index date (0, 1, 2, or ≥3), which we used as a surrogate marker for potential frontline workers or those with other risk factors for COVID-19.

The primary outcome was a positive RT-PCR test for SARS-CoV-2 infection within 21 days of the index date. We followed study subjects from the index date until the first occurrence of test positivity, death, end of the study period (May 27, 2021), or, in the unvaccinated group, vaccination.

We reported baseline demographic characteristics (age, sex, income quintile, rurality of region of residence, Public Health Unit of residence) for all individuals in the study cohort. We also examined past healthcare utilization (acute hospital admissions in the 3 years prior to index and number of outpatient physician visits in the 365 days prior to index), as well as the prevalence of various indicators of comorbidity, including chronic respiratory disease, chronic heart disease, hypertension, diabetes, autoimmune disease, chronic kidney disease, advanced liver disease, dementia, frailty, and history of stroke or transient ischemic attack (see supplemental tables for diagnosis codes used to define conditions). We used ecological variables from the 2016 Statistics Canada Census at the level of Dissemination Area (small, relatively stable geographic areas comprising 400–700 residents) to examine indicators of increased risk of SARS-CoV-2 infection (i.e., percentage of the population employed as essential workers, average number of people per household, percentage of the population without a diploma, percentage of the population not married, percentage of the population who self-identify as a visible minority, and percentage of the population that recently immigrated). Among vaccinated individuals, we identified the type of setting in which the vaccine was administered (congregate residential settings, hospital, mass vaccination centre, pharmacy, physician clinic, and other) as recorded in COVaxON. Congregate residential settings included supportive housing, homeless shelters, and other homes for special care. As noted, residents of nursing homes were excluded.

### Statistical analysis

Standardized differences based on the raw numbers were used to compare characteristics between groups with a standardized differences greater than 10% interpreted as meaningful differences between groups [8]. We used Cox proportional hazards models accounting for the matched nature of the data to estimate the association between receiving the first dose of a COVID-19 vaccine and SARS-CoV-2 infection within 21 days. The proportional hazards assumption was tested using a time-dependent covariate, and hazard ratios were calculated at 3, 7, 10, 14, 18, and 21 days. The primary analysis considered the entire cohort. Secondary analyses stratified by vaccination setting. All analyses were conducted at ICES using SAS version 9.4 (SAS Institute, Cary, NC) with the type 1 error rate set at 0.05 as the threshold for statistical significance.

## Results

We identified 4,798,430 Ontario residents who received their first dose of a COVID-19 vaccine between March 1, 2021 and May 6, 2021. After matching, the final cohort consisted of 4,744,007 (98.9%) matched pairs (**Table 1A and 1B**). Slightly more than half of study subjects (54.1%) were women, the average age was 59.2 years, and 87.4% of individuals had not been tested for SARS-CoV-2 in the 90 days preceding the index date. The exposure groups were similar across all measured covariates except for outpatient visits in the past year, with 42.6% of unvaccinated individuals having no outpatient physician visits in the past year compared to 34.8% of vaccinated individuals (standardized difference 0.16).

**Table 1:** A. Demographic characteristics of matched cohort. B. Characteristics of patient history among matched cohort.

**A**				
**Characteristic, n(%)**	**Total (N = 9,488,014)**	**Unvaccinated (N = 4,744,007)**	**Vaccinated (N = 4,744,007)**	**Standardized Differences**
**Baseline characteristics**				
Male	4,358,354 (45.9%)	2,179,177 (45.9%)	2,179,177 (45.9%)	0
Age, mean ± SD	59.03 ± 16.43	58.90 ± 16.39	59.16 ± 16.46	0.02
Neighbourhood income quintile				
1 (lowest)	1,626,143 (17.1%)	854,754 (18.0%)	771,389 (16.3%)	0.05
2	1,810,138 (19.1%)	917,605 (19.3%)	892,533 (18.8%)	0.01
3	1,912,224 (20.2%)	954,267 (20.1%)	957,957 (20.2%)	0
4	1,980,734 (20.9%)	976,781 (20.6%)	1,003,953 (21.2%)	0.01
5 (highest)	2,132,864 (22.5%)	1,026,939 (21.6%)	1,105,925 (23.3%)	0.04
Public Health Unit (PHU) region				
Central East	626,939 (6.6%)	314,359 (6.6%)	312,580 (6.6%)	0
Central West	1,753,684 (18.5%)	878,126 (18.5%)	875,558 (18.5%)	0
Durham	465,978 (4.9%)	232,644 (4.9%)	233,334 (4.9%)	0
Eastern	592,435 (6.2%)	296,730 (6.3%)	295,705 (6.2%)	0
North	545,189 (5.7%)	272,570 (5.7%)	272,619 (5.7%)	0
Ottawa	632,866 (6.7%)	316,559 (6.7%)	316,307 (6.7%)	0
Peel	970,382 (10.2%)	483,505 (10.2%)	486,877 (10.3%)	0
South West	1,092,913 (11.5%)	546,891 (11.5%)	546,022 (11.5%)	0
Toronto	1,907,394 (20.1%)	953,096 (20.1%)	954,298 (20.1%)	0
York	877,800 (9.3%)	437,571 (9.2%)	440,229 (9.3%)	0
Rural/small town	979,910 (10.3%)	491,802 (10.4%)	488,108 (10.3%)	0
Essential worker^a^	1,478,434 (15.6%)	766,460 (16.2%)	711,974 (15.0%)	0.03
Large household size	2,428,939 (25.6%)	1,209,630 (25.5%)	1,219,309 (25.7%)	0
Lower educational attainment^b^	1,453,484 (15.3%)	751,786 (15.8%)	701,698 (14.8%)	0.03
Not married	1,682,896 (17.7%)	880,323 (18.6%)	802,573 (16.9%)	0.04
Visible minority	2,483,628 (26.2%)	1,259,302 (26.5%)	1,224,326 (25.8%)	0.02
Recent immigrant	2,417,400 (25.5%)	1,233,964 (26.0%)	1,183,436 (24.9%)	0.02
**B**				
**Characteristic, n(%)**	**Total (N = 9,488,014)**	**Unvaccinated (N = 4,744,007)**	**Vaccinated (N = 4,744,007)**	**Standardized Differences**
**Baseline characteristics**				
Chronic respiratory disease	2,106,852 (22.2%)	1,028,142 (21.7%)	1,078,710 (22.7%)	0.03
Chronic heart disease	1,002,178 (10.6%)	484,441 (10.2%)	517,737 (10.9%)	0.02
Hypertension	3,739,536 (39.4%)	1,809,584 (38.1%)	1,929,952 (40.7%)	0.05
Diabetes	1,742,956 (18.4%)	856,629 (18.1%)	886,327 (18.7%)	0.02
Immunocompromised disorders	503,762 (5.3%)	234,155 (4.9%)	269,607 (5.7%)	0.03
Autoimmune disorders	460,069 (4.8%)	213,484 (4.5%)	246,585 (5.2%)	0.03
Chronic kidney disease	374,111 (3.9%)	178,084 (3.8%)	196,027 (4.1%)	0.02
Advanced liver disease	105,807 (1.1%)	53,338 (1.1%)	52,469 (1.1%)	0
Dementia	143,190 (1.5%)	73,316 (1.5%)	69,874 (1.5%)	0.01
Frailty	306,735 (3.2%)	154,101 (3.2%)	152,634 (3.2%)	0
History of stroke or transient ischemic attack	203,074 (2.1%)	102,521 (2.2%)	100,553 (2.1%)	0
Hospitalization in past 3 years				
0	8,491,003 (89.5%)	4,257,096 (89.7%)	4,233,907 (89.2%)	0.02
1	758,754 (8.0%)	368,687 (7.8%)	390,067 (8.2%)	0.02
2	158,995 (1.7%)	78,262 (1.6%)	80,733 (1.7%)	0
3+	79,262 (0.8%)	39,962 (0.8%)	39,300 (0.8%)	0
Outpatient physician visits in past 1 year				
0	3,672,070 (38.7%)	2,019,651 (42.6%)	1,652,419 (34.8%)	0.16
1	1,718,252 (18.1%)	816,570 (17.2%)	901,682 (19.0%)	0.05
2–4	2,435,151 (25.7%)	1,132,421 (23.9%)	1,302,730 (27.5%)	0.08
5–8	1,048,106 (11.0%)	483,566 (10.2%)	564,540 (11.9%)	0.05
9–14	433,360 (4.6%)	203,492 (4.3%)	229,868 (4.8%)	0.03
15+	181,075 (1.9%)	88,307 (1.9%)	92,768 (2.0%)	0.01
Number of prior SARS-CoV-2 tests				
0	8,293,814 (87.4%)	4,146,907 (87.4%)	4,146,907 (87.4%)	0
1	885,872 (9.3%)	442,936 (9.3%)	442,936 (9.3%)	0
2	166,732 (1.8%)	83,366 (1.8%)	83,366 (1.8%)	0
3+	141,596 (1.5%)	70,798 (1.5%)	70,798 (1.5%)	0

^a^ employed in sales, trades, manufacturing, agriculture.

^b^ employed in sales, trades, manufacturing, agriculture.

Vaccines were most often administered in public health mass vaccination clinics (N = 2,244,827; 47.3%) followed by hospital-based clinics (N = 1,134,734; 23.9%), pharmacies (N = 688,414; 14.5%), other settings (N = 486,113; 10.2%), physician offices (N = 143,522, 3.0%), and congregate residential settings (N = 46,397, 0.01%). Of the final cohort, 34,075 people (0.4%) had a laboratory-confirmed SARS-CoV-2 infection and 2,197,116 (23.2%) initially unvaccinated individuals received their first vaccine during the observation period.

In the primary analysis, the rate of SARS-CoV-2 infection was significantly lower among vaccine recipients vs non-recipients at all the post-vaccination time points we evaluated, except at 10 days: 3 days (adjusted HR 0.51; 95% CI 0.49 to 0.53), 7 days (HR 0.84; 95% CI 0.81 to 0.86), 14 days (HR 0.76; 95% CI 0.73 to 0.78), 18 days (HR 0.50; 95% CI 0.47 to 0.52), and 21 days (HR 0.36; 95% CI 0.34 to 0.39) **(Table 2)**. There was no difference in rate of SARS-CoV-2 infection between groups at 10 days (HR 1.01, 95% CI 0.97–1.04). Graphical representation of these hazard ratios over time can be found in the Appendix.

**Table 2:** Hazard ratios for the association between receipt of first dose of a COVID-19 vaccine and SARS-COV-2 infection according to time since vaccination and vaccination setting.

	Hazard Ratio (95% CI)					
Site	3 days	7 days	10 days	14 days	18 days	21 days
**Overall (all sites)**	0.51 (0.49–0.53)*	0.84 (0.81–0.86)*	1.01 (0.97–1.04)	0.76 (0.74–0.78)*	0.50 (0.47–0.52)*	0.36 (0.34–0.39)*
**Congregate Setting**	0.95 (0.60–1.49)	1.35 (1.00–1.83)*	1.49 (1.03–2.15)*	1.09 (0.81–1.47)	0.76 (0.48–1.20)	0.57 (0.30–1.11)
**Hospital-Based Clinic**	0.60 (0.55–0.66)*	0.90 (0.85–0.95)*	1.08 (1.00–1.16)*	0.84 (0.80–0.89)*	0.55 (0.50–0.59)*	0.39 (0.35–0.45)*
**Mass vaccination sites**	0.46 (0.43–0.49)*	0.82 (0.78–0.86)*	0.97 (0.92–1.02)	0.70 (0.67–0.73)*	0.44 (0.41–0.47)*	0.31 (0.28–0.35)*
**Pharmacy**	0.40 (0.35–0.46)*	0.68 (0.63–0.74)*	0.91 (0.83–1.01)	0.86 (0.79–0.93)*	0.64 (0.57–0.72)*	0.52 (0.43–0.62)*
**Physician office**	0.41 (0.29–0.58)*	0.74 (0.59–0.93)*	1.03 (0.78–1.36)	0.94 (0.75–1.18)	0.65 (0.48–0.87)*	0.49 (0.31–0.78)*
**Other**	0.60 (0.53–0.68)*	0.93 (0.85–1.02)	1.00 (0.90–1.11)	0.65 (0.60–0.71)*	0.38 (0.33–0.44)*	0.25 (0.20–0.31)*

*Statistically significant.

In the secondary analysis stratified by vaccination setting, results for mass vaccination clinics, pharmacies, and physician offices were consistent with the main findings. However, for individuals who received their first dose of a vaccine at a hospital-based clinic, there was a small but statistically significant increase in the rate of SARS-CoV-2 infection at 10 days (HR 1.08, 95% CI 1.00–1.16) compared to matched individuals who did not receive a vaccine. Similarly, individuals who received their first vaccine dose in congregate residential settings also had a higher rate of SARS-CoV-2 infection at 7 days (HR 1.35, 95% CI 1.00–1.83), and 10 days (HR 1.49, 95% CI 1.03–2.15) post-vaccination compared to their unvaccinated matched controls. At all other time points, the rate of SARS-CoV-2 infection was not significantly different between people vaccinated in congregate settings and their matched counterparts.

## Discussion

In this population-based cohort study that explored the early risk of SARS-CoV-2 infection after vaccination, we found a markedly low risk of early infection after vaccination in the cohort as a whole. However, among people vaccinated in congregate settings, we observed that the rate of SARS-CoV-2 infection was 35% and 49% greater in the 7 and 10 days after vaccination, respectively, compared to unvaccinated individuals. These results reinforce the overall safety of vaccine clinics, with no broad increase seen in the rate of infection in the period immediately after vaccination. However, the findings from congregate settings in particular suggest there may be a heightened risk of SARS-CoV-2 transmission in these settings that warrants continued vigilance, particularly as these are potentially people at highest risk of COVID complications.

The results of this study align with established clinical trial evidence regarding the onset of vaccine protection occurring within 7 days of the first dose [9,10]. Importantly, our findings support vaccine effectiveness at a population-level and counter early anecdotal observations of potential increased risk of infection at mass vaccination settings, which accounted for close to half of all vaccinated individuals in our study. These results support the continued use of many of these mechanisms for vaccination campaigns with no evidence of increased transmission of disease. Interestingly, the stronger protective effect in the initial 3 days after vaccine administration highlights that many of those electing to get vaccinated likely did so after following public health guidance regarding not presenting to vaccination sites with any COVID-related symptoms or recent exposure to people diagnosed with COVID-19. By the seventh day following receipt of the first vaccine dose, this selective protective effect was diminished, and re-aligns with expected estimates of vaccine effectiveness found in previous studies [7,9–12].

Despite the overall results, we did find an elevated rate of infection in the 7 to 10 days after vaccination in congregate settings. This aligns with the average incubation period of 5 days from time of infection and 1–2 days for testing after symptom presentation [13,14]. These results highlight the potential risk that continues to exist in congregate settings, such as group homes and homeless shelters, where some of the most vulnerable populations reside. The small but elevated risk for infection among those vaccinated in hospital-administered settings within 7 days is likely also associated with some hospital-led programs conducting outreach clinics for similar populations living in congregate settings. These findings may suggest transmission of SARS-CoV-2 from vaccinators to residents of these settings during the vaccination itself, or could be related to the standard processes for efficient vaccinating, where residents are gathered together in communal rooms to receive vaccine, increasing potential for airborne transmission between residents [15]. For example, in many programs when vaccines are administered at a group home or congregate living setting, all residents are brought together into a single congregate room with people from outside of the home, which may expedite any potential disease spread. Further exploration of means to ensure that vaccination clinics do not increase risk are important in this highly vulnerable population, specifically, those with socioeconomic vulnerability and those living with comorbidities that increase the risk of harm due to infection. Finally, these results may also be representative of the greater risk many of those living in congregate settings have given the inability for most to socially distance and the need to share space.

Some limitations of our study warrant discussion. First, the results derive from the early period of initial vaccine roll-out. Most individuals had not yet been vaccinated, and the generalizability to heavily vaccinated population mainly receiving booster doses is unknown. Although the chances of increased risk of disease transmission in congregate settings remain, we anticipate that the risks would be reduced due to greater population immunity and an increased awareness of airborne transmission. Importantly, with new variant it is unknown if the increase transmissibility would change these results and therefore ongoing work needed to assess potential risks of transmission when different variants are dominant. Second, we may not have identified all SARS-CoV-2 infections, particularly among those who were infected but remained asymptomatic and may therefore not have sought testing. However, we do not believe this risk would differ across sites. Finally, the groupings by which setting is reported in the COVaxON dataset precluded a more granular analysis of different vaccination settings. For example, hospital-organized community clinics or outreach events (e.g., mobile vaccine vans) would be categorized as hospital settings rather than a separate category [16].

## Conclusions

In this large population-based cohort of Ontario residents, we found that there was no increased risk of SARS CoV2 infection in the 14 days after first dose vaccination suggesting no broad transmission of disease at time of vaccination. However, some evidence of increased risk among those vaccinated in congregate settings, speaking to the need to consider opportunities for supporting safe vaccine administration in these settings where people typically gather at time of vaccination. Given ongoing booster immunization programs and potential future doses, our study highlights the need for continued vigilance during any mass vaccination processes and education regarding the delayed nature of protection following vaccination.

## Supporting information

S1 Fig Hazard ratios across time for all sites. (PPTX)

S2 Fig Hazard ratios across time, by vaccination site (A-F). (PPTX)

S1 File (DOCX)

S2 File (DOCX)
