# Supplementary figures and images for: Risk of SARS-CoV-2 infection following initial COVID-19 vaccination: Population-based cohort study
Source: PLoS One. 2022 Oct 20;17(10):e0273903. doi: 10.1371/journal.pone.0273903 (PMC9584446; doi:10.1371/journal.pone.0273903)

## Slide 1
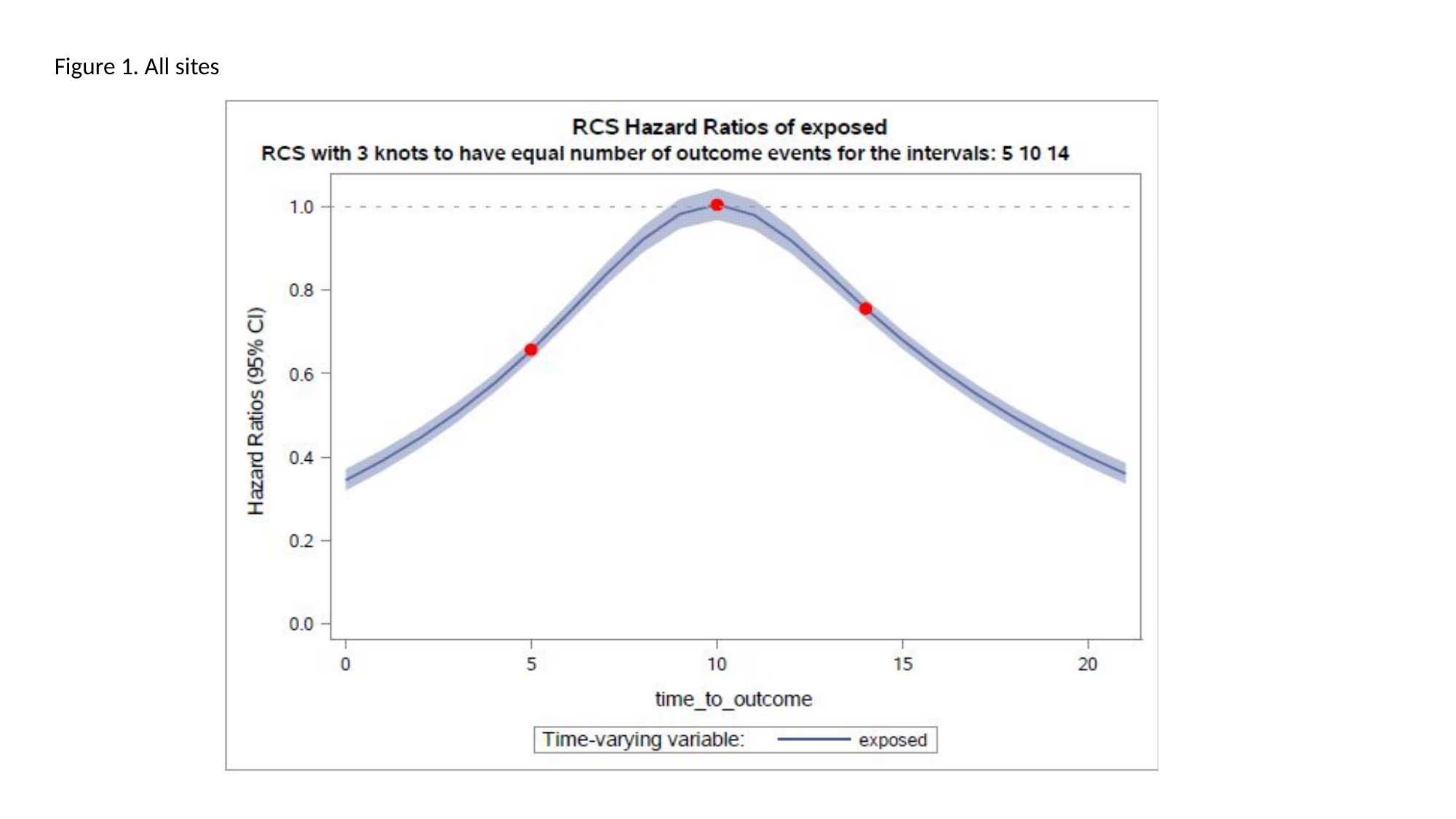

Figure 1. All sites

Supplement: S1 Fig — (PPTX) [file pone.0273903.s001.pptx]
