# Supplementary material for: Risk of SARS-CoV-2 infection following initial COVID-19 vaccination: Population-based cohort study
Source: PLoS One. 2022 Oct 20;17(10):e0273903. doi: 10.1371/journal.pone.0273903 (PMC9584446; doi:10.1371/journal.pone.0273903)
Supplement: S2 Fig — (PPTX) [file pone.0273903.s002.pptx]

## Slide 1
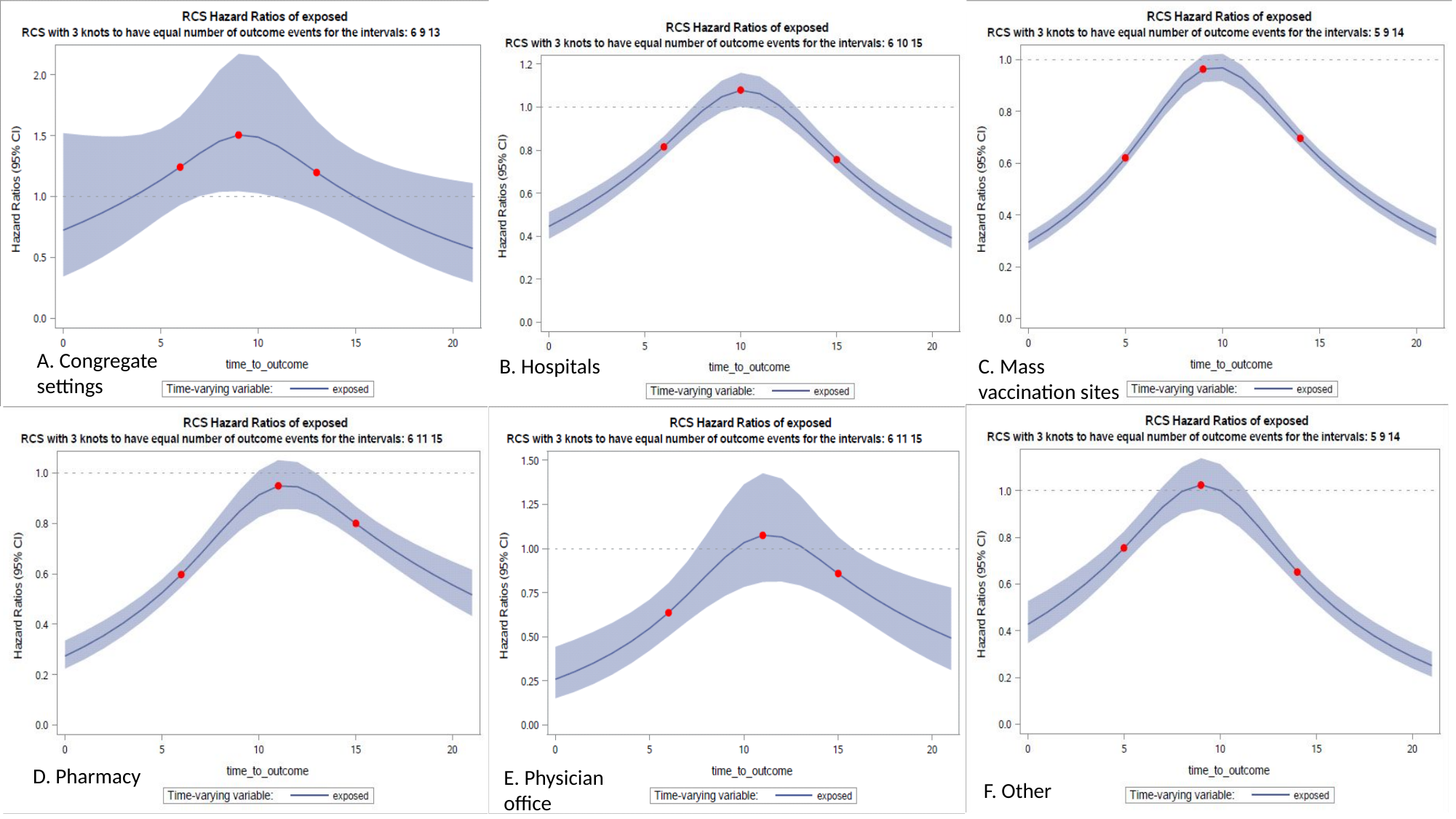

A. Congregate settings
B. Hospitals
C. Mass vaccination sites
D. Pharmacy
E. Physician office
F. Other
