# Supplementary material for: Risk of SARS-CoV-2 infection following initial COVID-19 vaccination: Population-based cohort study
Source: PLoS One. 2022 Oct 20;17(10):e0273903. doi: 10.1371/journal.pone.0273903 (PMC9584446; doi:10.1371/journal.pone.0273903)
Supplement: S2 File — (DOCX) [file pone.0273903.s004.docx]

**Supplemental Table 1. Diagnosis codes for comorbidities**

| **Disease/condition** | **Code (record must be prior to index date)** |
| --- | --- |
| Chronic respiratory disease | Asthma: Record in ICES ASTHMA database  COPD: Record in ICES COPD database |
| Congestive heart failure | Record in ICES CHF database |
| Hypertension | Record in ICES HYPER database |
| Diabetes | Record in ICES ODD database |
| Immunocompromised | HIV: Record in ICES HIV database |
|  | Transplant recipient (solid organ or stem cell):   - Solid organ transplant (CORR):   - For transplants before December 31, 2019: Use RECIPIENT_TREATMENT dataset in CORR library, and flag individuals where treatment_code=171   - For transplants on/after January 1, 2020: Look for ICD-10 dx, CCI procedure, and OHIP feecodes from DAD, NACRS and OHIP for RENAL, LIVER, LUNG, HEART, PANCREAS, INTESTINE - Allogenic/autologous bone marrow transplant (DAD, OHIP): - DAD: CCP (prcode) = 53.0; CCI (incode) = 1WY19, 1LZ19HHU7, 1LZ19HHU8 - OHIP: Feecode = Z426 - Immune system disorders in ACG: IMM_DIS flag in DAD, SDS, NACRS, OHIP with 2-year lookback - Sickle cell anemia: any hospitalization with any diagnosis code with ICD9: 282.6 or ICD10: D57.0-D57.2, D57.8 - Other immune system disorders: any hospitalization, ED visit or physician billing (DAD, NACRS, OHIP) with the following codes:   - DAD/NACRS: ICD-9 codes 273.2, 279.0, 279.1, 279.2, 279.3, 279.8, 279.9, 289.8 or ICD-10 codes D80, D81, D82, D83, D84, D89   - OHIP: dxcode = 279 - Immunosuppressive therapy   - >30 days (total days supplied) of oral corticosteroid in the 6 months before index   - Receipt of other immunocompromising drug (including antineoplastics) in the 6 months before index - Active cancer: Using the index date as the date to determine “active cancer” status, patient any of the following treatments in the past 6 months:   - Cancer surgery - CCI codes in DAD   - Radiation - source = Cancer and DX10CODE1 = Z510 in NACRS   - Chemotherapy - source = Cancer and DX10CODE1 = Z511 or Z512 in NACRS   - Any evidence of cancer diagnosis in OCR prior to last treatment date (surgery, radiation and chemotherapy).   - If not recent treatment for cancer, must have had a recent diagnosis – cancer diagnosis in OCR within the past year before index |
| Autoimmune disease | - Rheumatoid arthritis: record in ORAD - Inflammatory bowel disease: Record in OCCC - Psoriasis: 1 hospitalization or 3 physician billings   - DAD: ICD-9: 696.1, 696.8; ICD-10: L40.0, L40.1, L40.2, L40.3, L40.4, L40.8, L40.9   - OHIP: dxcode = 696 - Psoriatic arthritis: 1 hospitalization or (3 physician billings for psoriatic arthritis + 1 for psoriasis [696])   - DAD: ICD-9: 696.0; ICD-10: L40.5, M07.0, M07.1, M07.2, M07.3, M09.0   - OHIP: dxcode = 721 (at least one of these billings must be billed by a rheumatologist, where spec=48 [rheumatology]) - Multiple sclerosis: One hospitalization or 5 physician billings over 2 years with the following codes   - DAD: ICD-9: 340; ICD-10: G35   - OHIP: dxcode: 340 |
| Chronic kidney disease | Diagnostic as per ICES Concept Dictionary (see **Appendix A**), will be classified as CKD if meets either of the following:   - CKD diagnosis code in DAD, NACRS, OHIP in 5 years or - At least 1 dialysis code in each of the 3 months prior to index |
| Advanced liver disease | History of cirrhosis or decompensated cirrhosis using the following definitions in DAD, NACRS, OHIP (see **Appendix B** for codes used in Lapointe-Shaw et al. [2018])   - Cirrhosis: 2+ Outpatient or 1+ Inpatient CIRRHOSIS - Decompensated cirrhosis: 1+ Outpatient for CIRRHOSIS and (1+ inpatient diagnostic code OR 1+ procedure code) |
| Dementia/frailty | - Record in ICES DEMENTIA database - Medical conditions associated with frailty - Frailty flag in DAD, SDS, NACRS, OHIP with 2-year lookback |
| History of stroke or transient ischemic attack | Transient Ischemic Attack:  DAD and NACRS were used to identify patients with a history of a transient ischemic attack, based on at least 1 hospitalization or ED visit with a diagnosis coded with one of the following codes:  • ICD-9: 435, 3623  • ICD-10: G450, G451, G452, G453, G458, G459, H340  Acute Ischemic Stroke:  DAD was used to identify patients with a history of acute ischemic stroke, based on at least 1 hospitalization with a main diagnosis coded with one of the following codes:  • ICD-9: 434, 436  • ICD-10: I63 (excluding I63.6), I64, H34.1 |

**Supplemental Table 2. Chronic kidney disease and chronic dialysis codes from ICES Concept Dictionary:**

**Chronic Kidney Disease Codes**

International Classification of Disease (ICD) and OHIP diagnosis codes

| **SOURCE** | **CODE** | **DESCRIPTION** |
| --- | --- | --- |
| ICD-9 | 4030 | MAL HYPERTENS RENAL DIS W/ & W/OUT RF |
| ​ | 4031 | BEN HYPERTENS RENAL DISEASE W/ & W/OUT RF |
| ​ | 4039 | HYPERTENS RENAL DIS NOS W/ & W/OUT RF |
| ​ | 4040 | MAL HYPER HRT/REN DIS W/ & W/OUT RF & CHF |
| ​ | 4041 | BEN HYPER HRT/REN DIS W/ & W/OUT RF & CHF |
| ​ | 4049 | HYPER HRT/REN DIS NOS W/ & W/OUT RF & CHF |
| ​ | 585 | CHRONIC RENAL FAILURE |
| ​ | 586 | RENAL FAILURE NOS |
| ​ | 5888 | IMPAIRED RENAL FUNCT NEC |
| ​ | 5889 | IMPAIRED RENAL FUNCT NOS |
|  | 2504 | DIAB RENAL MANIF ADULT |
| ICD-10 | E102 | Type 1 diabetes mellitus with incipient diabetic nephropathy adequately or inadequately controlled with insulin, diet, oral agents |
| ​ | E112 | Type 2 diabetes mellitus with incipient diabetic nephropathy adequately or inadequately controlled with insulin, diet, oral agents |
| ​ | E132 | Other specified diabetes mellitus with incipient diabetic nephropathy adequately or inadequately controlled with insulin, diet, oral agents |
| ​ | E142 | Unspecified diabetes mellitus with incipient diabetic nephropathy adequately or inadequately controlled with insulin, diet, oral agents |
| ​ | I12 | Hypertensive renal disease |
| ​ | I13 | Hypertensive renal and heart disease |
| ​ | N08 | Glomerular disorders in diseases classified elsewhere |
| ​ | N18 | Chronic renal failure |
| ​ | N19 | Unspecified renal failure |
| OHIP dx | 403 | Hypertensive renal disease |
|  | 585 | Chronic renal failure, uremia |

Chronic Dialysis Codes:

(From DAD, SDS, OHIP)

| **Source** | **​Code** | **​Description** | **​Dialysis Type** |
| --- | --- | --- | --- |
| ​CCP | ​5195 | ​Hemodialysis | ​HD |
| ​ | ​6698* | ​Peritoneal dialysis | ​PD |
| ​CCI | ​1PZ21HQBS | ​Dialysis, urinary system NEC continuous venovenous hemodialysis | ​HD |
| ​ | ​1PZ21HQBR | ​Dialysis, urinary system NEC hemodialysis | ​HD |
| ​ | ​1PZ21HPD4 | ​Dialysis, urinary system NEC peritoneal dialysis using dialysate | ​PD |
| ​OHIP feecode | ​R849 | ​Dialysis – Heamodialysis - Initial & acute | ​HD |
| ​ | ​G323 | ​Dialysis – Haemodialysis - Acute, repeat (max 3) | ​HD |
| ​ | ​G325 | ​Dialysis – Haemodialysis - Medical component (incl in unit fee) | ​HD |
| ​ | G326 | ​Dialysis - Chronic, contin. haemodialysis or haemofiltration each | ​HD |
| ​ | ​G860 | ​Chronic hemodialysis  hospital location | ​HD |
| ​ | ​G862 | ​Hospital self-care chronic hemodialysis | ​HD |
| ​ | ​G863 | ​Chronic hemodialysis IHF location | ​HD |
| ​ | ​G865 | ​Chronic Home hemodialysis | ​Home HD |
| ​ | ​G866 | ​Intermittent hemodialysis treatment centre | ​HD |
| ​ | ​G082 | ​Continuous venovenous haemodialfiltration | ​HD |
| ​ | ​G083 | ​Continuous venovenous haemodialysis | ​HD |
| ​ | ​G085 | ​Continuous venovenous haemofiltration | ​HD |
| ​ | ​G090 | ​Veneovenous slow continuous ultrafiltration | ​HD |
| ​ | ​G091 | ​Continuous arteriovenous haemodialysis | ​HD |
| ​ | ​G092 | ​Continuous arteriovenous haemodiafiltration | ​HD |
| ​ | ​G093 | ​Haemodiafiltration - Contin. Init & Acute (repeatx3) | ​HD |
| ​ | ​G094 | ​Haemodiafiltration - Contin. Chronic | ​HD |
| ​ | ​G095 | ​Slow Continuous Ultra Filtration - Initial & Acute (repeat) | ​HD |
| ​ | ​G096 | ​Slow Continuous Ultra Filtration – Chronic | ​HD |
| ​ | ​G294 | ​Arteriovenous slow continuous ultrafiltration init and acute | ​HD |
| ​ | ​G295 | ​Continuous aterivenous haemofiltration initial and acute | ​HD |
| ​ | ​G330 | ​Peritoneal dialysis - Acute (up to 48 hrs) | ​PD |
| ​ | ​G331 | ​Peritoneal dialysis - Repeat acute (up to 48 hrs) max. 3 | ​PD |
| ​ | ​G332** | ​Peritoneal dialysis - Chronic (up to 48 hrs) | ​PD |
| ​ | ​G861 | ​Chronic peritoneal dialysis hospital location | ​PD |
| ​ | ​G864 | ​Chronic Home peritoneal dialysis | ​PD |
| ​ | ​G333 | ​Home/self-care dialysis | ​Both PD and Home HD |
| ​ | ​H540 | ​OOP Renal Dialysis Out-patient visit | ​All dialysis types |
| ​ | ​H740 | ​PRE-APPROVED OOC Out-patient Renal dialysis | ​All dialysis types |

(From CORR):

| **TREATMENT_CODE and Description** | **Dialysis Type** |
| --- | --- |
| 060 = 0 - Treatment-dependent Locations, 6 - PD combined with HD, 0 - Other​ | ​HD/PD |
| ​111 = 1 - Acute Care Hospital, 1 - Conventional HD, 1 - Total Care | ​HD |
| ​112 = 1 - Acute Care Hospital, 1 - Conventional HD, 2 - Limited Self Care | ​HD |
| ​113 = 1 - Acute Care Hospital, 1 - Conventional HD, 3 - Total Self Care | ​HD |
| ​121 = 1 - Acute Care Hospital, 2 - Short Daily HD, 1 - Total Care | ​HD |
| ​122 = 1 - Acute Care Hospital, 2 - Short Daily HD, 2 - Limited Self Care | ​HD |
| ​123 = 1 - Acute Care Hospital, 2 - Short Daily HD, 3 - Total Self Care | ​HD |
| ​131 = 1 - Acute Care Hospital, 3 - Slow Nocturnal HD, 1 - Total Care | ​HD |
| ​132 = 1 - Acute Care Hospital, 3 - Slow Nocturnal HD, 2 - Limited Self Care | ​HD |
| ​133 = 1 - Acute Care Hospital, 3 - Slow Nocturnal HD, 3 - Total Self Care | ​HD ​ |
| ​141 = 1 - Acute Care Hospital, 4  Continuous Ambulatory PD, 1 - Total Care | ​PD |
| ​151 = 1 - Acute Care Hospital, 5 - Automated PD, 1 - Total Care | ​PD |
| ​152 = 1 - Acute Care Hospital, 5 - Automated PD, 2 - Limited Self Care | ​PD |
| ​211 = 2 - Chronic Care Hospital, 1 - Conventional HD, 1 - Total Care | HD |
| ​221 = 2 - Chronic Care Hospital, 2 - Short Daily HD, 1 - Total Care | ​HD |
| ​231 = 2 - Chronic Care Hospital, 3 - Slow Nocturnal HD, 1 - Total Care | ​HD |
| ​241 = 2 - Chronic Care Hospital, 4 - Continuous Ambulatory PD, 1 - Total Care | ​PD |
| ​242 = 2 - Chronic Care Hospital, 4 - Continuous Ambulatory PD, 2 - Limited Self Care | ​PD |
| ​251 = 2 - Chronic Care Hospital, 5 - Automated PD, 1 - Total Care | ​PD |
| ​252 = 2 - Chronic Care Hospital, 5 - Automated PD, 2 - Limited Self Care | ​PD |
| ​311 = 3 - Community Centre, 1 - Conventional HD, 1 - Total Care | ​HD |
| ​312 = 3 - Community Centre, 1 - Conventional HD, 2 - Limited Self Care | ​HD |
| ​313 = 3 - Community Centre, 1 - Conventional HD, 3 - Total Self Care | ​HD |
| ​321 = 3 - Community Centre, 2 - Short Daily HD, 1 - Total Care | ​HD |
| ​322 = 3 - Community Centre, 2 - Short Daily HD, 2 - Limited Self Care | ​HD |
| ​323 = 3 - Community Centre, 2 - Short Daily HD, 3 - Total Self Care | ​HD |
| ​331 = 3 - Community Centre, 3 - Slow Nocturnal HD, 1 - Total Care | ​HD |
| ​332 = 3 - Community Centre, 3 - Slow Nocturnal HD, 2 - Limited Self Care | ​HD |
| ​333 = 3 - Community Centre, 3 - Slow Nocturnal HD, 3 - Total Self Care | ​HD |
| ​413 = 4 - Home, 1 - Conventional HD, 3 - Total Self Care | ​Home HD |
| ​423 = 4 - Home, 2 - Short Daily HD, 3 - Total Self Care | ​Home HD |
| ​433 = 4 - Home, 3 - Slow Nocturnal HD, 3 - Total Self Care | ​Home HD |
| ​443 = 4 - Home, 4 - Continuous Ambulatory PD, 3 - Total Self Care | ​PD |
| ​453 = 4 - Home, 5 - Automated PD, 3 - Total Self Care | ​PD |

**Supplemental Table 3. OHIP dxcodes, ICD-9 and 10 codes used to identify history of advance liver disease (cirrhosis and decompensated cirrhosis) (as per Lapointe-Shaw et al. [2018]):**

| **Cirrhosis** | |
| --- | --- |
| Physician visit code | OHIP: 571 |
| Hospital diagnostic codes | ICD-9: 456.1, 571.2, 571.5  ICD-10: I85.9, I98.2, K70.3, K71.7, K74.6 |
| **Decompensated Cirrhosis** | |
| Hospital diagnostic codes | ICD-9: 456.0, 456.2, 572.2, 572.3, 572.4, 782.4, 789.5  ICD-10: I85.0, I86.4, I98.20, I98.3, K721, K729, K76.6, K76.7, R17, R18 |
| Procedure codes | CCI: 1.NA.13.BA-FA, 1.NA.13.BA-X7, 1.NA.13.BA-BD, 1.KQ.76GP-NR, 1.OT.52.HA  CCP: 1006, 6691  OHIP: J057, Z591 |
